# Multimodal Blockade of the Renin–Angiotensin System in the Treatment of Cancer in Dogs Has Mild Adverse Effects in Some Dogs

**DOI:** 10.3390/vetsci11060275

**Published:** 2024-06-17

**Authors:** Keren E. Dittmer, Sarah Wetzel, Thomas Odom, John S. Munday, Elizabeth A. Flatt, Ingrid J. Wilson, Catherine Hughes, Swee T. Tan

**Affiliations:** 1 School of Veterinary Science, Massey University, Palmerston North 4410, New Zealand; k.e.dittmer@massey.ac.nz (K.E.D.);; 2 Vet South, Winton Clinic, Winton 9720, New Zealand; 3 Parkhill Vet Hospital, Birkenhead, Auckland 0626, New Zealand; 4 Shirley Vet Clinic, 15 Marshland Road, Shirley, Christchurch 8061, New Zealand; 5 Gillies McIndoe Research Institute, Wellington 7184, New Zealand; swee.tan@gmri.org.nz

**Keywords:** dog, renin–angiotensin system, cancer, osteosarcoma, melanoma, oncology, therapy

## Abstract

**Simple Summary:**

The renin–angiotensin system (RAS) is a well-known hormonal system that controls blood pressure and blood volume. Recent work has suggested that it also plays a role in cancer. Various studies in humans and animals have investigated blocking different parts of the RAS; many, but not all, studies have shown decreased tumor growth and spread. The RAS consists of various bypass pathways, which may explain the lack of a response to treatment in some studies. A treatment has been developed to simultaneously block multiple parts of the RAS (multimodal blockade). This treatment has been used in one clinical trial in humans with glioblastoma (a brain tumor) and another trial in cats with squamous cell carcinoma (skin cancer). The aim of this study was to assess the safety of multimodal blockade of the RAS in dogs with two different types of cancer (osteosarcoma and oral malignant melanoma). Two mild adverse effects were observed: one dog developed intermittent vomiting; another dog had a mildly increased serum SDMA concentration (a kidney function biomarker) at one time point. This sets the stage for conducting a larger-scale trial to assess the efficacy of this treatment for cancer in dogs.

**Abstract:**

The renin–angiotensin system (RAS) is increasingly being recognized to play a role in the tumor microenvironment, promoting tumor growth. Studies blocking a single part of the RAS have shown mixed results, possibly due to the existence of different bypass pathways and redundancy within the RAS. As such, multimodal blockade of the RAS has been developed to exert more complete inhibition of the RAS. The aim of the present study was to assess the safety of multimodal RAS blockade in dogs. Five dogs (four with appendicular osteosarcoma, one with oral malignant melanoma) were treated with atenolol, benazepril, curcumin, meloxicam, and metformin. The dogs underwent clinical examination, blood pressure measurement, and hematology and serum biochemistry tests performed at 0, 1, 3, 6, 9, and 12 weeks, then every 3 months thereafter. End-of-life decisions were made by the owners. None of the dogs developed hypotension. One dog had intermittent vomiting during the 64 weeks it was on the trial. One dog had a one-off increase in serum SDMA(symmetrical dimethylarginine) concentration. Dogs were euthanized at weeks 3 (osteosarcoma), 10 (osteosarcoma), 17 (osteosarcoma), and 26 (oral malignant melanoma), and one dog was still alive at the end of the trial at 64 weeks (osteosarcoma). This is the first assessment of multimodal blockade of the RAS in dogs, and the results suggest it causes only mild adverse effects in some animals. The efficacy of the treatment was not assessed due to the small number of dogs. This pilot study allows for future larger studies assessing multimodal RAS blockade for the treatment of canine cancer.

## 1. Introduction

The traditional role of the renin–angiotensin system (RAS) has been in the control of blood pressure and blood volume, in which prorenin is converted to renin, which in turn converts angiotensinogen to angiotensin I. Via the angiotensin-converting enzyme (ACE), angiotensin I is converted to angiotensin II, which activates the angiotensin II receptors [1]. Feeding into this pathway is cyclooxygenase-2 (COX-2), which can activate the prorenin receptor, resulting in the production of renin [2,3,4]. Cathepsin B and insulin-like growth factor can also increase renin production [3,4]. Chymase converts angiotensin I to angiotensin II. Additional peptides in RAS include angiotensin 2-8 heptapeptide (angiotensin III), angiotensin 3-8 hexapeptide (angiotensin IV), and angiotensin 1-7 heptapeptide [1].

More recently, the RAS has been found to play a role in the tumor microenvironment, with effects on tumor cells, hypoxia, angiogenesis, and inflammation, in addition to its role in stem cell regulation [5]. Numerous studies have looked at inhibiting different parts of the RAS, most commonly using an ACE inhibitor or angiotensin II type 1 receptor inhibitor. Results from these studies have been mixed. While some studies (mix of clinical trials, mouse models, and cell lines) have shown an increased risk of cancer, increased cell proliferation, or increased risk of metastasis [6,7,8], most studies have shown decreased tumor volume, decreased tumor growth, and decreased angiogenesis [9,10].

Cancer cells are notorious for being able to use alternative pathways, and the RAS has a high level of redundancy such that angiotensin II can be produced via classical and several alternative pathways. This could explain the variability in the response to the inhibition of the RAS in different trials; if only one part of the pathway is blocked, tumor cells could still utilize bypass pathways. To get around this phenomenon, treatments that block multiple parts of the RAS have been developed and have been used to treat humans with glioblastoma [11].

In this present study, a similar treatment to that used in human patients that simultaneously blocks multiple parts of the RAS activation (multimodal blockade) was assessed for safety. If this treatment was found to be well-tolerated, RAS blockade could be a potential treatment for canine cancer. The conversion of prorenin to renin is blocked using atenolol (a β-blocker that decreases renin production [12,13]), meloxicam (a COX2 inhibitor [14]), curcumin (a cathepsin B inhibitor [15]), and metformin (an inhibitor of the IGF-1 signaling pathway [16,17]). The conversion of angiotensin I to angiotensin II is blocked using the ACE inhibitor benazepril [18] and also curcumin (a chymase inhibitor) [19].

Only a few studies on the inhibition of the RAS as a treatment for cancer have been performed in dogs [20]. Losartan, an angiotensin II type 1 receptor antagonist, has been administered to dogs with metastatic osteosarcoma following prior treatment involving amputation and chemotherapy. This intervention results in a modest extension of progression-free survival [21]. Metformin has been applied to canine prostatic carcinoma and urothelial carcinoma cell lines, and has been shown to decrease cell proliferation and increase cell apoptosis [22]. Similarly, metformin decreases tumor growth in a xenografted metastatic canine mammary gland tumor cell line [23]. Metformin may have additional anti-cancer effects, other than via the inhibition of the RAS, as it also inhibits m-TOR (mammalian target of rapamycin) via the AMPK (AMP activated protein kinase)-dependent pathway, leading to cell growth arrest [20]. COX-2 inhibitors have been widely assessed in dogs for the treatment of cancer [24]. Piroxicam, deracoxib, firocoxib, and meloxicam, alone or with chemotherapy, have shown a generally positive effect on survival in dogs with urothelial carcinoma [24,25,26,27,28,29]. Piroxicam, in particular, has been evaluated in combination with chemotherapy for a number of canine cancers, including lymphoma, soft tissue sarcoma, squamous cell carcinoma, hemangiosarcoma, oral malignant melanoma, and osteosarcoma [30,31,32,33,34,35]. Curcumin has been shown to decrease cell proliferation in canine cell lines, including osteosarcoma, melanoma, mammary carcinoma, and mast cell tumors [36,37].

The multimodal blockade of the RAS has been assessed for safety in six cats [38]. In that study, cats with squamous cell carcinoma were treated for 8 weeks with the same medications as those used in the present study. No significant adverse effects on kidney or liver function and no hypotension were observed in that study.

The aim of this study was to assess the safety of multimodal blockade of the RAS in dogs. Five dogs, four with osteosarcoma and one with oral malignant melanoma, were enrolled in this study and closely monitored for any adverse effects. Owing to the small number of dogs in the trial, the response of the neoplasms to treatment was unable to be evaluated.

## 2. Materials and Methods

### 2.1. Study Design

This study was a proof of concept, open-label, pilot safety evaluation to investigate any potential side effects of multimodal blockade of the RAS in dogs. To minimize potential side effects, dogs received a step-wise increase in drug doses over a 3-week period as per Table 1. At the beginning of week 3 of the trial, the dogs were at the dose rate at which they would continue for the entirety of the trial. The final dose rates were as follows: meloxicam at 0.1 mg/kg q 24 h (Ilium meloxicam 1.5 mg/mL, Troy Laboratories Pty Ltd., Glendenning, NSW, Australia), curcumin (NHV turmeric 160–180 mg curcumin/mL dietary supplement, NHV Natural Pet Products, Vancouver, BC, Canada, 7–13 kg 1 mL, 14–20 kg 1.5 mL, 21–27 kg 2 mL, 28–34 kg 2.5 mL, and >24 kg 3.0 mL), metformin at 10 mg/kg q 12 h (Metformin 500 mg, Apotex NZ Ltd., Auckland, New Zealand), atenolol at 0.25 mg/kg q 12 h (Mylan atenolol 50 mg, Mylan NZ Ltd., Auckland, New Zealand), and benazepril at 0.25 mg/kg q 24 h (VetACE benazepril 20 mg, Jurox Animal Health, Auckland, New Zealand or Apex benazepril oral solution 5 mg/mL, Dechra Veterinary Products, Somersby, NSW, Australia). The dose rates were based on label inserts or the literature (in the case of metformin [39]). Doses were calculated for each animal, and tablets were cut and weighed to provide the precise dosage.

### 2.2. Animals

Dogs were enrolled in the study if they had a confirmed histologic diagnosis of oral malignant melanoma or osteosarcoma. Cases of osteosarcoma had to be treated with amputation, with or without adjuvant chemotherapy. For inclusion in the trial, cases of oral malignant melanoma had to either be inoperable or the owner declined surgery, and radiation was not available. Dogs were excluded if they had signs of obvious ill health, systolic blood pressure of less than 120 mm Hg, or any sign of kidney disease at week 0.

To monitor treatment, each dog underwent a physical examination, blood pressure assessment, a complete blood count, and serum biochemistry as per the study protocol shown in Table 2. In cases 1, 2, 4, and 5, blood pressure was measured using an oscillometric blood pressure monitor, while in case 3, blood pressure was measured using Doppler ultrasonography. Additionally, owners answered questions regarding any changes noted in their animals, such as vomiting, diarrhea, lethargy, and how easy it was to administer the medications.

Decisions on end-of-life were determined by the owner in consultation with their veterinarian. If euthanized, dogs underwent a full postmortem examination with samples taken of any recurrent or remaining tumor, metastases, other lesions, and samples of the lung, liver, heart, kidney, brain, stomach, small intestine, colon, pancreas, and spleen, to assess any potential toxicity. Routine histologic analysis was performed on all samples.

## 3. Results

Five dogs were recruited into the study, four with appendicular osteosarcoma and one with oral malignant melanoma. Details of the dogs, diagnosis, time in trial, and outcome are included in Table 3.

Four out of the five dogs in the study were euthanized due to tumor progression, while one dog was still alive at the end of the trial, after 64 weeks of treatment. The results of the postmortem examination of the four dogs euthanized are included in Table 4. All lesions found at postmortem examination were attributable to tumor progression or pre-existing disease, with no evidence of toxicity in any of the organs examined.

The monitoring of the dogs during the study showed no detrimental impacts of the treatment on weight or blood pressure. Blood pressure and selected analytes from the complete blood count and serum biochemistry are presented in Table 5. Serum ALT and ALP activity were increased in Case 2 at week 0 and week 1. These increases, coupled with clinical signs, like a distended abdomen, sparse hair coat, and enlarged cranial organs, initially suggested hyperadrenocorticism. However, due to the poor prognosis associated with osteosarcoma, further diagnostic confirmation was not pursued. Case 3 exhibited an increase in serum SDMA (symmetric dimethylarginine) concentration to 21 μg/dL (reference range 0–15 μg/dL) in week 2. At week 3, the serum SDMA concentration had returned to normal. However, in week 6, the serum SDMA concentration increased again to 16 μg/dL. Based on the table of adverse events in the Veterinary Cooperative Oncology Group—Common Terminology Criteria for Adverse Events (VCOG-CTCAE v2), the SDMA at week 2 would be a grade 2 adverse event (SDMA between 18–25 μg/dL), and that at week 6 would be a grade 1 adverse event (SDMA between 16–18 μg/dL) [40]. However, these grades are for when SDMA is persistently greater than the stated values and urine specific gravity is less than 1.030. SDMA was not persistently increased in case 3 and, unfortunately, a urine sample was not obtained.

The owners of the dogs did not report any problems administering the medications, with the exception of two owners, one administered the medications in cheese, the other in peanut butter. Two owners reported that their dogs seemed particularly fond of the curcumin medication.

Except for the days just prior to euthanasia, the owners did not report any lethargy, decrease in appetite, or change in the behavior of their dog. With the exception of case 4, the dogs did not exhibit any vomiting or diarrhea. Case 4, however, developed vomiting in week 1, vomiting 2–3 times per week initially, peaking at 8 times per week in week 4 when the metformin dose reached 10 mg/kg. Sucralfate 1 g q 12 h was started to alleviate the vomiting. At week 6, case 4 was vomiting 3–4 times per week. The vomiting continued, peaking again in week 19 at 4–5 times per week. At this point, the metformin dose was dropped to 7.5 mg/kg and famotidine at 0.5 mg/kg q 12 h was started. Two weeks later, the metformin dose was dropped again to 5 mg/kg (this dose was continued for the remainder of the trial), famotidine was continued, and after this, the vomiting appeared to decrease in frequency. Variable vomiting was reported thereafter, ranging from 1–2 times a day to no vomiting for several weeks, but never completely resolved. During all this time, case 4 was reported to be “doing well”, eating normally, energetic, and playing fetch. According to the VCOG-CTCAE this would be consistent with a grade 2 event, where the event is moderate and outpatient noninvasive intervention is indicated [40]. However, this grade is associated with moderate limitation of the activities of daily living (eating, drinking, sleeping, defecating, and urination), and this was not reported to be the situation for case 4. It was considered probable that the vomiting was likely related to one of the medications [40], most likely metformin, given that vomiting is a reported side effect of this drug.

Samples of the osteosarcomas and oral malignant melanoma from the four dogs euthanized were examined histologically. A summary of the histology results for the dogs is presented in Table 6. Case 5 had an oral malignant melanoma, which pre-trial had a nuclear atypia score of 5, 28 mitoses per 2.37 mm^2^, and a total tumor score of 35 [41]. After euthanasia, case 5 had a nuclear atypia score of 5, 42 mitoses per 2.37 mm^2^, and a total tumor score of 42 [41]. Other than tumor metastases, no histologic abnormalities were detected in other organs, with the exception of case 2, who had adrenal hyperplasia and hepatocellular vacuolation consistent with probable pituitary-dependent hyperadrenocorticism.

## 4. Discussion

When evaluating a novel treatment for cancer in animals, it is important to determine if adverse effects occur that either impact the quality of life of the animal or that are detrimental to its health. All the medications used in this study have been previously studied in dogs, although not their use in combination. Thus, the purpose of this study was to determine if multimodal treatment resulted in adverse side effects. The results of this study show that multimodal blockade of the RAS did result in mild adverse effects that probably resulted from the therapy, most notably vomiting and an increase in serum SDMA concentration.

Given the role of the RAS in blood pressure homeostasis, it was considered that hypotension would be the most likely side effect of the treatment. However, this did not appear to be the case for the five dogs in the trial. Normal blood pressure in dogs ranges from 110 to 160 mmHg for systolic pressure and 60 to 90 mmHg for diastolic pressure [44]. Blood pressure in all dogs was generally within the normal range or hypertensive, and while it increased and decreased from the baseline at different points, there was no clear downward trend in blood pressure. Certainly, many dogs had measurements that would be considered severely hypertensive (>180 mmHg); however, this was likely situational hypertension, whereby excitement or anxiety results in increased blood pressure [44]. Additionally, blood pressure is usually 10–20 mmHg higher in greyhounds compared with other breeds [45,46]. However, we cannot rule out that the situational hypertension was masking mild hypotension.

Case 3 exhibited a one-off increase in serum SDMA concentration in week 2, which was considered a grade 2 or moderate adverse effect based on VCOG-CTCAE v2 guidelines, although a urine sample was not obtained, so it is unknown as to whether urine specific gravity was less than 1.030 [40]. Differentials for a single increase in the serum SDMA concentration include recovery from mild injury and compensatory mechanisms. Despite the high blood pressure measured in-clinic, it is possible that a period of hypotension could have caused mild kidney injury [47,48] and the increase in the serum SDMA concentration. It should be noted that this dog was a greyhound, a breed which has been previously reported to have higher serum SDMA concentrations than other dog breeds [49,50]. One study determined a reference interval for serum SDMA concentration in greyhounds of 6.3–19.9 ug/dL [49]. Using this reference interval, the increase in the serum SDMA concentration in week 2 would be more consistent with a grade 1 mild adverse event, and the serum SDMA concentration in week 12 would be within normal limits. Additionally, it has been shown that some cats and dogs with cancer, particularly lymphoma, may have increased serum SDMA concentrations that are not associated with concomitant increases in serum creatinine concentration, as in case 3 [51]. Hypotheses for the increase include microscopic invasion of the kidney by cancer cells and the production of SDMA by tumor cells [51]. The latter is highly plausible as the expression of SDMA by cancer cells is associated with increased cell survival, and human osteosarcoma cell lines have been shown to produce SDMA [52]. The serum SDMA concentrations in dogs with osteosarcoma have not been assessed, but it is possible that the increases in SDMA in case 3 were associated with the tumor, rather than kidney disease. Therefore, further research on the utility of the serum SDMA concentration for assessing kidney function in animals with cancer is required.

The other adverse effect reported in this study was vomiting. Gastrointestinal clinical signs, such as vomiting, diarrhea, and inappetence, are reported adverse effects of metformin administration in dogs [39,53]. In order to minimize the likelihood of gastrointestinal clinical signs, the dose rate of metformin used in the present trial was increased in 2.5 mg/kg increments over a period of 3 weeks, as previously described in a canine trial of metformin [39]. Despite this, one dog exhibited regular vomiting which started after week 1 and peaked when the metformin dose rate peaked. Despite decreasing the dose rate of metformin to 5 mg/kg and including medications to decrease gastrointestinal irritation, intermittent vomiting continued for the entire time for which this dog was in the trial. However, at the same time as the dog started multimodal therapy, it was also receiving carboplatin treatment for osteosarcoma, and vomiting is a reported side effect of carboplatin treatment [54]. As such, the combination of medications could have made gastrointestinal signs more likely. Nonetheless, intermittent vomiting continued beyond the completion of the carboplatin protocol and for the duration of the 64-week trial. Due to the timing of the vomiting, which is a known adverse effect of metformin, the vomiting was probably attributed to the treatment. The owner reported that, despite the vomiting, the dog remained bright and playful and showed no signs of lethargy or inappetence. Therefore, it appears that the quality of life was minimally impacted by the vomiting. Further research on whether concurrent chemotherapy potentiates the gastrointestinal clinical signs of metformin is required before the widespread concurrent use of multimodal blockade of the RAS.

While this pilot study was not designed to assess whether the treatment had any impact on survival, the survival times reported in this study are similar to those reported for osteosarcoma and oral malignant melanoma in the literature [41,55,56]. In a similar study using multimodal blockade of the RAS as a treatment for squamous cell carcinoma in cats, it was noted that the oral and nasal planum neoplasms had unusually low mitotic rates for this type of cancer [38]. Unfortunately, in that study, the mitotic rate of the tumor prior to starting the trial was unknown, which, along with the small number of cats in the trial, made the interpretation of the mitotic rate results difficult. In this present study, the mitotic rate (number of mitoses in a 2.37 mm^2^ field) was assessed prior to starting the trial and postmortem. In two osteosarcoma cases (cases 1 and 3), there was a decrease in the mitotic rate (from 46 to 11, and 21 to 12), while in the oral malignant melanoma case, the mitotic rate increased (from 28 to 42). Even though cases 1 and 3 had a decreased mitotic rate, both cases had metastases and the tumor grade was unaffected in case 3, so it is unclear whether the decrease in the mitotic rate had any impact on survival. To fully assess the influence of multimodal blockade of the RAS on survival, a randomized blinded controlled clinical trial with larger numbers would be required.

Osteosarcoma and oral malignant melanoma were the canine neoplasms of choice in this study due to the poor survival time for these neoplasms and, in the case of oral malignant melanoma and axial osteosarcoma, sometimes limited ability to provide surgical treatment. If a new non-chemotherapy-based treatment using affordable off-patent repurposed medications could successfully improve survival time for these neoplasms, then this would be a major breakthrough. Additionally, experimental evidence in human osteosarcoma cell lines suggests that suppressing RAS signaling decreases cell viability, migration, and invasion, as well as increases cell apoptosis [57]. Similarly, in human melanoma cell lines, blockade of the angiotensin II receptor type 2 results in the inhibition of cell growth and angiogenesis [8]. As such, it was reasonable to expect that multimodal blockade of the RAS may have a beneficial effect on these tumors. However, blockade of angiotensin II receptor type 1 results in increased cell growth, suggesting different roles for the different angiotensin receptors in melanoma [8], which could explain the increased mitotic rate seen in case 5 with oral malignant melanoma. It may be that multimodal RAS blockade needs to be more selective for different types of receptors in some tumors, rather than blocking the entire system. Further research on the suitability of different canine tumors for multimodal RAS blockade is required.

One of the important features of any potential treatment in animals is the ease of administration of that treatment. Multimodal blockade of RAS required the administration of five medications, three of which were twice daily, including two to three liquids and two to three tablets (depending on the size of the dog). This required significant commitment on the part of the owners. To make administration easier for the owners, each tablet was pre-cut and weighed to ensure the correct dosage; this decreased the amount of time and effort for each owner, but was a time-consuming part of trial management. Three out of five of the owners had no problems administering any of the medications, however, one owner had to hide the medications in cheese, and another in peanut butter. In future trials using multimodal blockade of the RAS, it would be advantageous to develop a palatable combined long-acting formulations—tablet, paste, or liquid—to make administration of the medication by owners easier and allow more efficient use of time in trial management, particularly when expanded out to clinical practice. While the owners in the trial were extremely committed and they reported that all medications were administered, it cannot be ruled out that some doses might have been missed, and any impact this could have had on the trial is unknown. Evidence suggests that owners may over-report medication administration compliance when self-reporting [58]. Additionally, because direct measurement of the drug concentration in the blood was not performed in this trial, it is not possible to be completely sure as to the safety and efficacy of the drugs used in this combination, which is another limitation of this study. In future trials, serial blood collection and measurement of drug concentrations would enable this information to be obtained. Moreover, a further limitation of this study is the small number of dogs included. Future trials should assess the safety and efficacy of multimodal blockade of the RAS in a greater number of dogs.

## 5. Conclusions

While two adverse events were noted, they were mild in nature, and only vomiting was probably associated with the treatment. Clients should be warned of the possibility of vomiting as a potential side effect of the treatment in future trials. Additionally, while blood pressure was not adversely affected, an increased serum SDMA concentration was noted at two separate time points in one animal; as such, this should be closely monitored in any future larger trial. Microscopically, two dogs with osteosarcoma had a marked decrease in mitotic rate between the initial biopsy and at postmortem examination; further study is required to determine if this has an effect on survival. The results of this pilot study allow future larger studies assessing multimodal RAS blockade for the treatment of canine cancer.

## Figures and Tables

**Table 1:** Drug doses by week for multimodal blockage of the renin–angiotensin system in dogs.

Week	Meloxicam	Curcumin	Metformin	Atenolol	Benazepril
0	0.1 mg/kg q 24 h	Half label for wgt q 12 h	2.5 mg/kg q 12 h	0.125 mg/kg q 12 h	Not given
1	0.1 mg/kg q 24 h	Full label for wgt q 12 h	5 mg/kg q 12 h	0.25 mg/kg q 12 h	0.125 mg/kg q 24 h
2	0.1 mg/kg q 24 h	Full label for wgt q 12 h	7.5 mg/kg q 12 h	0.25 mg/kg q 12 h	0.25 mg/kg q 24 h
3+	0.1 mg/kg q 24 h	Full label for wgt q 12 h	10 mg/kg q 12 h	0.25 mg/kg q 12 h	0.25 mg/kg q 24 h

**Table 2:** Protocol for dogs on the multimodal blockade of the renin–angiotensin system trial.

Week	Physical Exam	Blood Pressure	CBC/Biochem ^1^
0	X	X	X
1	X	X	X
2	X	X	n.d.
3	X	X	X
6	X	X	X
9	X	X	X
12	X	X	X
3 monthly thereafter	X	X	X

^1^ CBC/Biochem = Complete blood count and serum biochemistry; n.d. = not done, X = sampling occurred.

**Table 3:** Signalment, weight, clinical information, weeks in trial, and outcome of trial.

Case	Age, Sex, Breed	Weight	Diagnosis	Surgery	Other Medications	Weeks	Outcome
1	11 yo FS Greyhound	25 kg	Osteosarcoma femur	Hind quarter amputation	None administered	10	Euthanized
2	12 yo MN Terrier cross	10 kg	Osteosarcoma scapula	Fore quarter amputation	None administered	3	Euthanized
3	5 yo MN Greyhound	32 kg	Osteosarcoma radius	Fore quarter amputation	Gabapentin, amoxicillin–clavulanic acid (week 0–4)	17	Euthanized
4	5 yo MN Rottweiler	38 kg	Osteosarcoma radius	Fore quarter amputation	Carboplatin, gabapentin, acetaminophen, famotidine, sucralfate	64	Still alive at end of trial
5	8 yo F Heading dog	21 kg	Oral malignant melanoma	None	Gabapentin, amantadine	26	Euthanized

F = female, S = spayed, M = male, N = neutered.

**Table 4:** Results of postmortem examination of dogs in the trial that were euthanized.

Case	Results of Postmortem Examination
1	Fractured humerus due to osteosarcoma, tumor recurrence at hind limb amputation site
2	Tumor recurrence at amputation site, invasion into spinal column and thoracic cavity, hemothorax, lung metastases
3	Tumor recurrence at amputation site and metastases to lung, liver, brain, heart, endocardiosis
5	Extensive invasion through palate into nasal cavity, metastases to mandibular, retropharyngeal, popliteal, inguinal, and mediastinal lymph nodes, and kidney, liver, bone, and lung

**Table 5:** Results of blood pressure monitoring and selected blood analytes until euthanized or the first 12 weeks.

Case	Week	Mean SP/DP (MAP)	HCT	Creat	SDMA	ALT	ALP
1	0	200/98 (153)	0.6	129	11	49	45
	1	137/81 (128)	0.59	109	11	63	42
	2	178/113 (134)	n.d.	n.d.	n.d.	n.d.	n.d.
	3	208/138 (166)	0.61	117	9	27	29
	6	173/135 (135)	0.62	115	10	38	48
	9	167/116 (136)	0.63	121	11	46	61
2	0	207/121 (151)	0.40	62	15	101 H	256 H
	1	168/84 (112)	0.38	50	8	79 H	336 H
	2	138/75 (95)	n.d.	n.d.	n.d.	n.d.	n.d.
	3	150/81 (103)	n.d.	n.d.	n.d.	n.d.	n.d.
3	0	n.d.	0.53	117	9	30	42
	1	121 (SP)	0.58	109	10	35	26
	2	120 (SP)	0.57	95	21 H	33	30
	3	n.d.	0.55	134	11	30	41
	6	173 (SP)	0.54	121	12	26 L	37
	9	186 (SP)	0.56	118	13	27 L	40
	12	150 (SP)	0.53	126	16 H	35	57
4	0	138/70 (97)	0.44	86	6	22	30
	1	142/75 (102)	0.46	84	5	31	39
	2	127/68 (92)	n.d.	n.d.	n.d.	n.d.	n.d.
	3	130/76 (97)	0.45	102	13	22	46
	6	130/79 (100)	0.5	99	6	32	48
	9	126/66 (86)	0.48	80	9	32	44
	12	127/80 (96)	0.42	96	9	16	41
5	0	227/122 (156)	0.41	66	12	46	34
	1	200/115 (143)	0.45	68	10	45	41
	2	206/126 (154)	n.d.	n.d.	n.d.	n.d.	n.d.
	3	247/109 (160)	n.d.	n.d.	n.d.	n.d.	n.d.
	6	189/122 (144)	0.42	58	10	45	51
	9	141/105 (119)	0.39	59	9	32	71
	12	192/138 (156)	0.34	52 L	9	37	54

SP = systolic pressure (mmHg); DP = diastolic pressure (mmHg); MAP = mean arterial pressure (mmHg); HCT = hematocrit (reference range 0.37–0.55 L/L; 0.49–0.65 L/L greyhound specific range); creat = creatinine (reference range 53–123 μmol/L; 88–150 μmol/L greyhound specific range); SDMA = symmetric dimethylarginine (0–14 μg/dL); ALT = alanine amino transferase activity (0–75 IU/L; 28–82 IU/L greyhound specific range); ALP = alkaline phosphatase activity (0–185 IU/L); n.d. = not done.

**Table 6:** Summary of neoplasm histology results pre-trial and postmortem.

Case	Pre/Post Trial	Tumor Subtype	Grade A ^1^	Grade B ^2^	Mitoses in 2.37 mm^2^
1	Pre	Osteoblastic productive	n.d.	n.d.	46
	Postmortem	Osteoblastic productive	I	I	11
2	Pre	Osteoblastic productive	III	II	36
	Postmortem	Osteoblastic productive	III	II	36
3	Pre	Osteoblastic productive	II	II	21
	Postmortem	Osteoblastic productive	II	II	12
4	Pre	Osteoblastic productive	n.d.	n.d.	26
5	Pre	Epitheloid malignant melanoma	n.a.	n.a.	28
	Postmortem	Epitheloid malignant melanoma	n.a.	n.a.	42

^1^ Grade A—based on grading system by Loukopoulos and Robinson 2007 [42]; ^2^ Grade B—based on grading system by Kirpensteijn et al. 2002 [43]; n.d. = not done; n.a. = not applicable.

## Data Availability

The data presented in this study are contained within the article.
